# 3-Amino­carbonyl­pyridinium difluoro­acetate at 123 K

**DOI:** 10.1107/S1600536809043414

**Published:** 2009-10-28

**Authors:** Julie Bardin, Alastair J. Florence, Jean-Baptiste Arlin, Alan R. Kennedy, Li Ven Wong

**Affiliations:** a Strathclyde Institute of Pharmacy and Biomedical Sciences, The John Arbuthnott Building, University of Strathclyde, 27 Taylor Street, Glasgow G4 0NR, Scotland; b WestCHEM, Department of Pure & Applied Chemistry, University of Strathclyde, 295 Cathedral Street, Glasgow G1 1XL, Scotland

## Abstract

In the crystal of the title compound, C_6_H_7_N_2_O^+^·C_2_HF_2_O_2_^−^, the cation adopts a catemeric N—H⋯O hydrogen-bonded chain motif involving the carboxamide group, with two further N—H⋯O hydrogen bonds connecting the cations to adjacent difluoro­acetate anions *via* the carboxamide and pyridinium N atoms. The carboxamide group of the nicotinamidium ion is twisted by 32.3 (6)° from the pyridine ring plane. A number of C—H⋯O and C—H⋯F interactions consolidate the packing.

## Related literature

For nicotinamide, see: Wright & King (1954 ▶); Miwa *et al.* (1999 ▶); Hino *et al.* (2001 ▶). For nicotinamide solvates, co-crystals and salts, see: Bardin *et al.* (2009 ▶); Koman *et al.* (2003 ▶); Athimoolam & Natarajan (2007*a* ▶,*b* ▶); Fleischman *et al.* (2003 ▶); Berry *et al.* (2008 ▶). Identification was initially made using multi-sample foil transmission X-ray powder diffraction analysis, see: Florence *et al.* (2003 ▶).
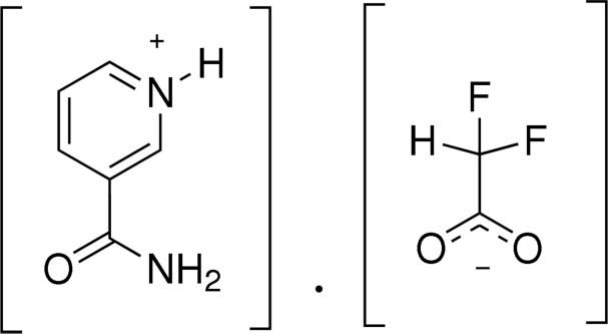


## Experimental

### 

#### Crystal data

C_6_H_7_N_2_O^+^·C_2_HF_2_O_2_^−^
*M* *_r_* = 218.16
Monoclinic, 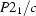

*a* = 4.9888 (2) Å
*b* = 25.6147 (12) Å
*c* = 7.2006 (4) Å
β = 105.912 (2)°
*V* = 884.88 (7) Å^3^
*Z* = 4
Mo *K*α radiation
μ = 0.15 mm^−1^
*T* = 123 K
0.30 × 0.10 × 0.02 mm

#### Data collection

Bruker APEXII CCD diffractometer
Absorption correction: multi-scan (*SADABS*; Sheldrick, 2002 ▶) *T*_min_ = 0.929, *T*_max_ = 0.997
8921 measured reflections
2201 independent reflections
2087 reflections with *I* > 2σ(*I*)
*R*_int_ = 0.015

#### Refinement

*R*[*F* ^2^ > 2σ(*F* ^2^)] = 0.034
*wR*(*F* ^2^) = 0.091
*S* = 1.05
2201 reflections
152 parameters
1 restraint
H atoms treated by a mixture of independent and constrained refinement
Δρ_max_ = 0.41 e Å^−3^
Δρ_min_ = −0.31 e Å^−3^

### 

Data collection: *APEX2* (Bruker, 2007 ▶); cell refinement: *SAINT* (Bruker, 2007 ▶); data reduction: *SAINT*; program(s) used to solve structure: *SHELXS97* (Sheldrick, 2008 ▶); program(s) used to refine structure: *SHELXL97* (Sheldrick, 2008 ▶); molecular graphics: *PLATON* (Spek, 2009 ▶) and *Mercury* (Macrae *et al.*, 2006 ▶); software used to prepare material for publication: *PLATON*.

## Supplementary Material

Crystal structure: contains datablocks global, I. DOI: 10.1107/S1600536809043414/fl2272sup1.cif

Structure factors: contains datablocks I. DOI: 10.1107/S1600536809043414/fl2272Isup2.hkl

Additional supplementary materials:  crystallographic information; 3D view; checkCIF report

## Figures and Tables

**Table 1:** Hydrogen-bond geometry (Å, °)

*D*—H⋯*A*	*D*—H	H⋯*A*	*D*⋯*A*	*D*—H⋯*A*
N1—H1*N*⋯O2^i^	0.920 (12)	2.589 (17)	3.2275 (12)	127.0 (13)
N1—H1*N*⋯O3^i^	0.920 (12)	1.675 (12)	2.5921 (12)	174.3 (17)
N2—H2*N*⋯O1^ii^	0.895 (16)	2.099 (15)	2.9801 (12)	167.9 (13)
N2—H3*N*⋯O3	0.863 (15)	2.046 (15)	2.8836 (11)	163.2 (13)
C1—H1⋯O1^i^	0.95	2.30	3.1763 (13)	154
C3—H3⋯F1^iii^	0.95	2.53	3.1645 (13)	124
C4—H4⋯F2^iv^	0.95	2.50	3.2885 (13)	141
C5—H5⋯O2^iv^	0.95	2.40	3.2458 (13)	149
C8—H6⋯O2^ii^	0.960 (14)	2.405 (15)	3.2403 (14)	145.2 (11)
C8—H6⋯O2^v^	0.960 (14)	2.577 (13)	3.3558 (13)	138.3 (12)

Symmetry codes: (i) 
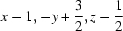
; (ii) 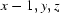; (iii) 
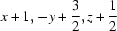
; (iv) 
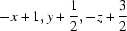
; (v) 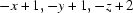.

**Fig. 1.**
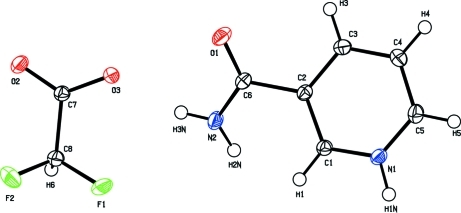
View of the title compound with the atom numbering scheme. Displacement ellipsoids for non-H atoms are drawn at the 50% probability level.

**Fig. 2.**
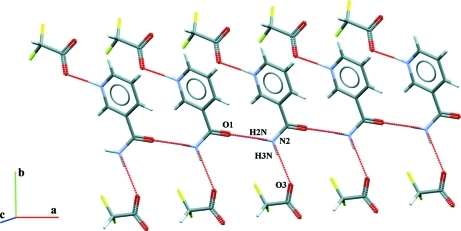
The NA+ cations form chain motifs via N2—H2N···O1 interactions between carboxamide groups with two further N—H···O contacts formed to the DFA- anions.

**Fig. 3.**
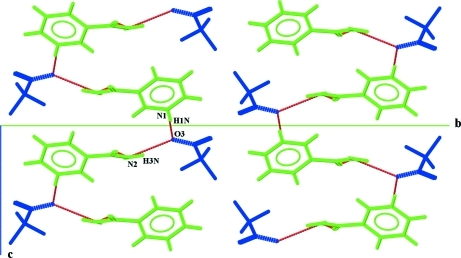
Chains of NA+ cations propagate down the a-axis with atom O3 connecting adjacent catemeric chains of cations (shown end on) via two N—H···O hydrogen bonds to N2—H3N on the carboxamide and N1—H1N on the pyridinium ring.

## supplementary crystallographic information

### Comment

Nicotinamide (C_6_H_6_N_2_O, NA) is a member of the vitamin B group that includes pyridoxine, riboflavin and thiamine and its crystal structure was first determined in 1954 (Wright & King, 1954). Additional experimental investigations of charge density (Miwa *et al.*,1999) and polymorphism (Hino *et al.*, 2001) have also been reported although the crystal structures of other polymorphic forms have not been published. A number of multicomponent crystalline forms have been investigated including a trifluoroethanol solvate (Bardin *et al.*, 2009), 3,5-dinitrosalicylate (Koman *et al.*, 2003), 2*R*,3*R*-tartrate hydrate (Athimoolam & Natarajan, 2007*a*) and trifluoroacetate (Athimoolam & Natarajan, 2007*b*) salts. Co-crystals of NA have also been reported with drugs such as carbamazepine (Fleischman *et al.*, 2003), salicylic acid and both the racemic and single enantiomer (S(+)) forms of ibuprofen (Berry *et al.*, 2008).

The difluoroacetate (DFA^-^) salt reported here (Scheme 1) was discovered during a study of multicomponent crystal formation involving fluorinated solvents and a range of organic compounds. The ions in the NA^+^ DFA^-^ salt crystallize in space group *P*2_1_/c with one NA^+^ cation and one DFA^-^ anion in the asymmetric unit (Fig.1). The internal C1—N1—C5 angle of the pyridinium ring in NA^+^ DFA^-^ is 122.13 (9)°, in close agreement to the value reported in the NA^+^ TFA^-^ salt (122.5 (4)°) but represents a significant increase over the the non-ionized form of NA (117.59 (7)°, Miwa *et al.*, 1999). In the DFA^-^ salt, as in the TFA^-^ salt and NA structures, the carboxamide group is not coplanar with the pyridinium ring with the angle between the planes of these groups being 32.30 (6)° [16.3 (8)° and 22.16 (4)° for NA^+^ TFA^-^ and NA respectively]. The packing in NA^+^ DFA^-^ consists of hydrogen bonded chains of NA^+^ ions extending parallel to the *a*-axis *via* contact N2—H2N···O1 between the anti-oriented H atom of the NH_2_ group and the carbonyl O-atom, O1 (Fig. 2). Each cation forms an N—H···O hydrogen bond to O3 on a DFA^-^ ion and this atom also accepts a second N—H···O hydrogen bond from a neighbouring cation in a parallel chain (Figure 3), that connect adjacent chains resulting in a two-dimensional sheet lying parallel to the *ac* plane. The remaining O-atom on the DFA^-^ ion, O2, whilst not involved in any hydrogen bonds is involved in three C—H···O contacts to two DFA^-^ ions and a cation. One further C—H···O contact is observed between O1 and C1—H1 on the pyridinium ring in a parallel chain of cations. C—H···F contacts are also observed (C3—H3···F1 and C4—H4···F2, see Table 2).

### Experimental

The novel structure reported here was discovered during a study of multicomponent crystal formation in organic compounds using a range of fluorinated acids. Identification of the novel phase was initially made using multi-sample foil transmission X-ray powder diffraction analysis (Florence *et al.*, 2003) and a suitable single-crystal for structure determination was obtained by isothermal evaporation at 278 K of a saturated solution of nicotinamide in DFAA.

### Refinement

Aromatic H atoms bound to C were placed in idealized positions and in a riding mode, with C—H distance set to 0.95Å and *U*_iso_ equal to 1.2 times *U*_eq_ of the parent atom. All other H atoms were located by difference synthesis and then refined isotropically.

### Crystal data

**Table:**

C_6_H_7_N_2_O^+^·C_2_HF_2_O_2_^−^	*F*(000) = 448
*M**_r_* = 218.16	*D*_x_ = 1.638 Mg m^−^^3^
Monoclinic, *P*2_1_/*c*	Mo *K*α radiation, λ = 0.71073 Å
Hall symbol: -P 2ybc	Cell parameters from 6311 reflections
*a* = 4.9888 (2) Å	θ = 2.9–28.4°
*b* = 25.6147 (12) Å	µ = 0.15 mm^−^^1^
*c* = 7.2006 (4) Å	*T* = 123 K
β = 105.912 (2)°	Slab, colourless
*V* = 884.88 (7) Å^3^	0.30 × 0.10 × 0.02 mm
*Z* = 4	

### Data collection

**Table:**

Bruker APEXII CCD diffractometer	2201 independent reflections
Radiation source: fine-focus sealed tube	2087 reflections with *I* > 2σ(*I*)
graphite	*R*_int_ = 0.015
φ and ω scans	θ_max_ = 28.4°, θ_min_ = 1.6°
Absorption correction: multi-scan (*SADABS*; Sheldrick, 2002)	*h* = −6→6
*T*_min_ = 0.929, *T*_max_ = 0.997	*k* = −32→34
8921 measured reflections	*l* = −8→9

### Refinement

**Table:**

Refinement on *F*^2^	Primary atom site location: structure-invariant direct methods
Least-squares matrix: full	Secondary atom site location: difference Fourier map
*R*[*F*^2^ > 2σ(*F*^2^)] = 0.034	Hydrogen site location: inferred from neighbouring sites
*wR*(*F*^2^) = 0.091	H atoms treated by a mixture of independent and constrained refinement
*S* = 1.05	*w* = 1/[σ^2^(*F*_o_^2^) + (0.0508*P*)^2^ + 0.323*P*] where *P* = (*F*_o_^2^ + 2*F*_c_^2^)/3
2201 reflections	(Δ/σ)_max_ = 0.001
152 parameters	Δρ_max_ = 0.41 e Å^−^^3^
1 restraint	Δρ_min_ = −0.31 e Å^−^^3^

### Special details

**Table:**

Geometry. Bond distances, angles *etc*. have been calculated using the rounded fractional coordinates. All su's are estimated from the variances of the (full) variance-covariance matrix. The cell e.s.d.'s are taken into account in the estimation of distances, angles and torsion angles
Refinement. Refinement on *F*^2^ for ALL reflections except those flagged by the user for potential systematic errors. Weighted *R*-factors *wR* and all goodnesses of fit *S* are based on *F*^2^, conventional *R*-factors *R* are based on *F*, with *F* set to zero for negative *F*^2^. The observed criterion of *F*^2^ > σ(*F*^2^) is used only for calculating -*R*-factor-obs *etc*. and is not relevant to the choice of reflections for refinement. *R*-factors based on *F*^2^ are statistically about twice as large as those based on *F*, and *R*-factors based on ALL data will be even larger.

### Fractional atomic coordinates and isotropic or equivalent isotropic displacement parameters (Å^2^)

**Table:**

	*x*	*y*	*z*	*U*_iso_*/*U*_eq_	
O1	0.80371 (15)	0.74427 (3)	0.79966 (12)	0.0214 (2)	
N1	0.11038 (17)	0.87200 (3)	0.62567 (12)	0.0159 (2)	
N2	0.36157 (18)	0.71520 (3)	0.75584 (14)	0.0186 (3)	
C1	0.19020 (19)	0.82177 (4)	0.64341 (14)	0.0146 (2)	
C2	0.44978 (19)	0.80807 (4)	0.76136 (14)	0.0139 (2)	
C3	0.6222 (2)	0.84727 (4)	0.86318 (15)	0.0176 (3)	
C4	0.5320 (2)	0.89865 (4)	0.84480 (15)	0.0199 (3)	
C5	0.2730 (2)	0.91037 (4)	0.72202 (15)	0.0187 (3)	
C6	0.5522 (2)	0.75269 (4)	0.77430 (14)	0.0149 (2)	
F1	0.12172 (14)	0.59643 (3)	0.66362 (11)	0.0300 (2)	
F2	0.27896 (15)	0.52181 (3)	0.59164 (10)	0.0292 (2)	
O2	0.73632 (16)	0.53207 (3)	0.90515 (13)	0.0238 (3)	
O3	0.61539 (15)	0.61630 (3)	0.89351 (11)	0.0201 (2)	
C7	0.56993 (19)	0.56809 (4)	0.85520 (14)	0.0147 (2)	
C8	0.2715 (2)	0.55331 (4)	0.74290 (15)	0.0177 (3)	
H1	0.06810	0.79550	0.57480	0.0180*	
H1N	−0.063 (2)	0.8784 (7)	0.543 (2)	0.045 (5)*	
H2N	0.185 (3)	0.7234 (6)	0.750 (2)	0.026 (4)*	
H3	0.80110	0.83880	0.94500	0.0210*	
H3N	0.422 (3)	0.6835 (6)	0.773 (2)	0.027 (4)*	
H4	0.64680	0.92550	0.91570	0.0240*	
H5	0.21060	0.94550	0.70600	0.0220*	
H6	0.171 (3)	0.5363 (5)	0.822 (2)	0.017 (3)*	

### Atomic displacement parameters (Å^2^)

**Table:**

	*U*^11^	*U*^22^	*U*^33^	*U*^12^	*U*^13^	*U*^23^
O1	0.0136 (4)	0.0203 (4)	0.0305 (4)	0.0040 (3)	0.0065 (3)	0.0053 (3)
N1	0.0135 (4)	0.0148 (4)	0.0183 (4)	0.0022 (3)	0.0027 (3)	0.0026 (3)
N2	0.0156 (4)	0.0124 (4)	0.0281 (5)	0.0020 (3)	0.0066 (3)	0.0021 (3)
C1	0.0130 (4)	0.0137 (4)	0.0168 (4)	−0.0002 (3)	0.0034 (3)	0.0009 (3)
C2	0.0132 (4)	0.0132 (4)	0.0154 (4)	0.0012 (3)	0.0042 (3)	0.0020 (3)
C3	0.0140 (4)	0.0181 (5)	0.0186 (4)	0.0002 (3)	0.0011 (3)	0.0005 (3)
C4	0.0196 (5)	0.0156 (5)	0.0223 (5)	−0.0022 (4)	0.0023 (4)	−0.0023 (4)
C5	0.0202 (5)	0.0139 (4)	0.0218 (5)	0.0017 (3)	0.0054 (4)	0.0009 (3)
C6	0.0144 (4)	0.0152 (4)	0.0149 (4)	0.0025 (3)	0.0039 (3)	0.0021 (3)
F1	0.0183 (3)	0.0226 (3)	0.0402 (4)	0.0048 (2)	−0.0072 (3)	0.0045 (3)
F2	0.0318 (4)	0.0254 (3)	0.0267 (4)	−0.0055 (3)	0.0020 (3)	−0.0094 (3)
O2	0.0151 (4)	0.0154 (4)	0.0377 (5)	0.0025 (3)	0.0019 (3)	0.0020 (3)
O3	0.0158 (3)	0.0128 (3)	0.0276 (4)	−0.0002 (3)	−0.0007 (3)	−0.0019 (3)
C7	0.0122 (4)	0.0140 (4)	0.0175 (4)	−0.0006 (3)	0.0036 (3)	0.0005 (3)
C8	0.0146 (4)	0.0146 (4)	0.0216 (5)	−0.0006 (3)	0.0011 (4)	0.0003 (4)

### Geometric parameters (Å, °)

**Table:**

F1—C8	1.3676 (13)	C1—C2	1.3851 (14)
F2—C8	1.3642 (13)	C2—C6	1.5020 (14)
O1—C6	1.2364 (13)	C2—C3	1.3928 (14)
O2—C7	1.2272 (13)	C3—C4	1.3854 (15)
O3—C7	1.2721 (13)	C4—C5	1.3838 (15)
N1—C1	1.3425 (13)	C1—H1	0.9500
N1—C5	1.3405 (13)	C3—H3	0.9500
N2—C6	1.3323 (13)	C4—H4	0.9500
N1—H1N	0.920 (12)	C5—H5	0.9500
N2—H2N	0.895 (16)	C7—C8	1.5342 (14)
N2—H3N	0.863 (15)	C8—H6	0.960 (14)
			
F1···C3^i^	3.1645 (13)	C1···O1^xi^	3.1802 (13)
F1···O3	2.6133 (11)	C1···N2^iii^	3.2748 (14)
F1···C4^i^	3.1997 (13)	C1···O1^i^	3.1763 (13)
F2···C4^ii^	3.2885 (13)	C1···O3^i^	3.3389 (13)
F2···C4^iii^	3.1834 (13)	C3···N1^vii^	3.3972 (14)
F2···F2^iv^	2.9535 (11)	C3···F1^vi^	3.1645 (13)
F2···F2^v^	3.0741 (11)	C4···F2^x^	3.1834 (13)
F2···O2	2.7472 (11)	C4···O3^iii^	3.4089 (13)
F2···C5^iii^	3.1716 (13)	C4···F2^xii^	3.2885 (13)
F1···H4^i^	2.6100	C4···F1^vi^	3.1997 (13)
F1···H3^i^	2.5300	C5···O2^i^	3.3464 (14)
F1···H3N	2.682 (15)	C5···C8^iii^	3.5712 (15)
F2···H4^iii^	2.8400	C5···F2^x^	3.1716 (13)
F2···H5^iii^	2.8300	C5···O3^iii^	3.3459 (13)
F2···H4^ii^	2.5000	C5···C7^iii^	3.4089 (14)
O1···C1^vi^	3.1763 (13)	C5···O2^xii^	3.2458 (13)
O1···C1^vii^	3.1802 (13)	C7···C5^x^	3.4089 (14)
O1···N2^vii^	2.9801 (12)	C7···N1^vi^	3.2463 (13)
O2···C8^vii^	3.2403 (14)	C8···C5^x^	3.5712 (15)
O2···F2	2.7472 (11)	C8···O2^ix^	3.3558 (13)
O2···O2^viii^	3.0848 (12)	C8···O2^xi^	3.2403 (14)
O2···C8^ix^	3.3558 (13)	C1···H2N^iii^	3.054 (14)
O2···C5^ii^	3.2458 (13)	C1···H2N	2.636 (15)
O2···N1^vi^	3.2275 (12)	C7···H3N	3.065 (15)
O2···C5^vi^	3.3464 (14)	C7···H1N^vi^	2.388 (14)
O3···F1	2.6133 (11)	H1···O1^i^	2.3000
O3···C5^x^	3.3459 (13)	H1···H2N	2.2200
O3···N2	2.8836 (11)	H1···N2	2.6500
O3···N1^vi^	2.5921 (12)	H1···O1^xi^	2.6900
O3···C1^vi^	3.3389 (13)	H1N···O3^i^	1.675 (12)
O3···C4^x^	3.4089 (13)	H1N···C7^i^	2.388 (14)
O1···H2N^vii^	2.099 (15)	H1N···O2^i^	2.589 (17)
O1···H3	2.6400	H2N···C1^x^	3.054 (14)
O1···H1^vi^	2.3000	H2N···O1^xi^	2.099 (15)
O1···H1^vii^	2.6900	H2N···C1	2.636 (15)
O2···H6^ix^	2.577 (13)	H2N···H1	2.2200
O2···H1N^vi^	2.589 (17)	H3···O1	2.6400
O2···H6^vii^	2.405 (15)	H3···F1^vi^	2.5300
O2···H5^vi^	2.7900	H3N···F1	2.682 (15)
O2···H5^ii^	2.4000	H3N···O3	2.046 (15)
O3···H3N	2.046 (15)	H3N···C7	3.065 (15)
O3···H1N^vi^	1.675 (12)	H4···F2^x^	2.8400
N1···C3^xi^	3.3972 (14)	H4···F2^xii^	2.5000
N1···C7^i^	3.2463 (13)	H4···F1^vi^	2.6100
N1···O2^i^	3.2275 (12)	H5···F2^x^	2.8300
N1···O3^i^	2.5921 (12)	H5···O2^i^	2.7900
N2···O3	2.8836 (11)	H5···O2^xii^	2.4000
N2···O1^xi^	2.9801 (12)	H6···O2^xi^	2.405 (15)
N2···C1^x^	3.2748 (14)	H6···O2^ix^	2.577 (13)
N2···H1	2.6500		
			
C1—N1—C5	122.13 (9)	C2—C1—H1	120.00
C1—N1—H1N	115.7 (11)	C2—C3—H3	120.00
C5—N1—H1N	122.2 (11)	C4—C3—H3	120.00
C6—N2—H2N	120.1 (10)	C3—C4—H4	120.00
H2N—N2—H3N	121.9 (14)	C5—C4—H4	120.00
C6—N2—H3N	116.9 (10)	N1—C5—H5	120.00
N1—C1—C2	120.28 (9)	C4—C5—H5	120.00
C1—C2—C6	121.49 (9)	O2—C7—O3	126.89 (10)
C1—C2—C3	118.61 (9)	O2—C7—C8	116.72 (9)
C3—C2—C6	119.85 (9)	O3—C7—C8	116.32 (9)
C2—C3—C4	119.80 (10)	F1—C8—F2	106.01 (8)
C3—C4—C5	119.28 (10)	F1—C8—C7	111.16 (8)
N1—C5—C4	119.88 (9)	F2—C8—C7	109.38 (8)
O1—C6—C2	119.22 (9)	F1—C8—H6	107.4 (9)
N2—C6—C2	116.95 (9)	F2—C8—H6	109.8 (8)
O1—C6—N2	123.83 (10)	C7—C8—H6	112.9 (9)
N1—C1—H1	120.00		
			
C5—N1—C1—C2	−1.18 (15)	C3—C2—C6—O1	−30.80 (14)
C1—N1—C5—C4	−0.15 (15)	C3—C2—C6—N2	149.70 (10)
N1—C1—C2—C3	1.24 (15)	C2—C3—C4—C5	−1.27 (15)
N1—C1—C2—C6	−176.03 (9)	C3—C4—C5—N1	1.38 (15)
C1—C2—C3—C4	−0.02 (15)	O2—C7—C8—F1	−168.25 (9)
C6—C2—C3—C4	177.30 (9)	O2—C7—C8—F2	−51.53 (12)
C1—C2—C6—O1	146.44 (10)	O3—C7—C8—F1	14.57 (12)
C1—C2—C6—N2	−33.06 (14)	O3—C7—C8—F2	131.29 (9)

Symmetry codes: (i) *x*−1, −*y*+3/2, *z*−1/2; (ii) −*x*+1, *y*−1/2, −*z*+3/2; (iii) *x*, −*y*+3/2, *z*−1/2; (iv) −*x*, −*y*+1, −*z*+1; (v) −*x*+1, −*y*+1, −*z*+1; (vi) *x*+1, −*y*+3/2, *z*+1/2; (vii) *x*+1, *y*, *z*; (viii) −*x*+2, −*y*+1, −*z*+2; (ix) −*x*+1, −*y*+1, −*z*+2; (x) *x*, −*y*+3/2, *z*+1/2; (xi) *x*−1, *y*, *z*; (xii) −*x*+1, *y*+1/2, −*z*+3/2.

### Hydrogen-bond geometry (Å, °)

**Table:**

*D*—H···*A*	*D*—H	H···*A*	*D*···*A*	*D*—H···*A*
N1—H1N···O2^i^	0.920 (12)	2.589 (17)	3.2275 (12)	127.0 (13)
N1—H1N···O3^i^	0.920 (12)	1.675 (12)	2.5921 (12)	174.3 (17)
N2—H2N···O1^xi^	0.895 (16)	2.099 (15)	2.9801 (12)	167.9 (13)
N2—H3N···O3	0.863 (15)	2.046 (15)	2.8836 (11)	163.2 (13)
C1—H1···O1^i^	0.95	2.30	3.1763 (13)	154
C3—H3···F1^vi^	0.95	2.53	3.1645 (13)	124
C4—H4···F2^xii^	0.95	2.50	3.2885 (13)	141
C5—H5···O2^xii^	0.95	2.40	3.2458 (13)	149
C8—H6···O2^xi^	0.960 (14)	2.405 (15)	3.2403 (14)	145.2 (11)
C8—H6···O2^ix^	0.960 (14)	2.577 (13)	3.3558 (13)	138.3 (12)

Symmetry codes: (i) *x*−1, −*y*+3/2, *z*−1/2; (xi) *x*−1, *y*, *z*; (vi) *x*+1, −*y*+3/2, *z*+1/2; (xii) −*x*+1, *y*+1/2, −*z*+3/2; (ix) −*x*+1, −*y*+1, −*z*+2.
